# Creating Consumer-Generated Health Data: Interviews and a Pilot Trial Exploring How and Why Patients Engage

**DOI:** 10.2196/12367

**Published:** 2019-06-13

**Authors:** Kara Burns, Craig A McBride, Bhaveshkumar Patel, Gerard FitzGerald, Shane Mathews, Judy Drennan

**Affiliations:** 1 QUT Business School Queensland University of Technology Brisbane Australia; 2 Queensland Children's Hospital Children's Health Queensland Brisbane Australia; 3 School of Public Health & Social Work Queensland University of Technology Brisbane Australia

**Keywords:** patient generated health data, patient engagement, patient participation, mHealth, photography

## Abstract

**Background:**

Consumer-generated health data (CGHD) are any clinically relevant data collected by patients or their carers (consumers) that may improve health care outcomes. Like patient experience measures, these data reflect the consumer perspective and is part of a patient-centric agenda. The use of CGHD is believed to enhance diagnosis, patient engagement, and thus foster an improved therapeutic partnership with health care providers.

**Objective:**

The aim of this study was to further identify how these data were used by consumers and how it influences engagement via a validated framework. In addition, carer data has not been explored for the purpose of engagement.

**Methods:**

Study 1 used interviews with CGHD-experienced patients, carers, and doctors to understand attitudes about data collection and use, developing an ontological framework. Study 2 was a pilot trial with carers (parents) of children undergoing laparoscopic appendectomy. For 10 days carers generated and emailed surgical site photographs to a tertiary children’s hospital. Subsequently, carers were interviewed about the engagement framework. In total, 60 interviews were analyzed using theme and content analysis.

**Results:**

This study validates a framework anchored in engagement literature, which categorizes CGHD engagement outcomes into 4 domains: physiological, cognitive, emotional, and behavioral. CGHD use is complex, interconnected, and can be organized into 10 themes within these 4 domains.

**Conclusions:**

CGHD can instigate an ecosystem of engagement and provide clinicians with an enhanced therapeutic relationship through an extended view into the patient’s world. In addition to clinical diagnosis and efficient use of health care resources, data offer another tool to manage consumers service experience, especially the emotions associated with the health care journey. Collection and use of data increases consumers sense of reassurance, improves communication with providers, and promotes greater personal responsibility, indicating an empowering consumer process. Finally, it can also improve confidence and satisfaction in the service.

## Introduction

Consumer-generated health data (CGHD) is any clinically relevant data, collected and controlled by a patient or carer, to be used in the health care setting [1]. It has been dubbed the “blockbuster drug of the century” [2] because of the speculation that CGHD will promote engagement [3]. Research indicates CGHD improves quality of life [4], promotes health behaviors [5], increases considerations of service value and satisfaction [6,7], and has the potential to increase patient empowerment [8]. CGHD can be distinguished from patient-reported outcome measures, which are typically managed by providers [9], although both are now recognized to promote patient engagement and enhance care through monitoring and assessing symptoms, informing treatment decisions, tracking outcomes, agenda setting, and enhancing patient-provider communication [10].

CGHD is primarily useful to health care professionals in the diagnosis and management of conditions. Data can be clinician requested (solicited) or patient initiated (unsolicited) and are aggregated in many forms [1]. Quantitative measures of spirometry, qualitative descriptions such as a diary of emotions, and visual information such as photographs of dietary intake are common [11], with data capture encouraged through mobile phone apps and wearable technology. This research focuses on consumer-generated photographs that are useful for rheumatology [12], dermatology [4], surgical documentation [13], and wound management [14].

Research on CGHD has predominantly focused on benefits for the health care professional. The use and engagement outcomes promoted by the data for the patient and carer is still under-researched [15]. Previous studies show that data improves patient activation [16], a state characterized by an informed, knowledgeable, active patient who can sustain a course of health care under pressure [17]. Tang et al (2006) [16] suggest patients could experience a better quality of life and Frühauf et al (2012) [4] report this in a study of tele-dermatology where “all patients perceived they had made savings of time and expenses, and moreover, they believed they had gained a more flexible and empowered lifestyle.” Tan et al (2012) conducted a qualitative interview study based on perceptions of unsolicited photographs. Notably, general practitioners also believed the images could empower patients, saying it helped patients retain control and “the patient became more part of the team [8].” In addition, carers are critical to engagement in some patient groups [18]; thus, their perspective is also valuable to explore.

A recent systematic review of patient engagement found that of the 89 randomized controlled trials that purported to instigate and measure engagement, 21 of those had no quantifiable measure. Of the 10 high-quality papers elected for review, only 1 study defined engagement a-priori [19]. Little research has evaluated the effect of CGHD on engagement, and an ontological framework for describing and measuring patient engagement is absent in extant literature [20]. Given the importance of patient engagement, the growing evidence of its ability to improve health outcomes, defining engagement is critical.

Using the education engagement literature, this study defines engagement as composed of physiological (clinical), cognitive, emotional, and behavior dimensions [21]. Engagement was chosen because it is a multifaceted, situation-specific concept, which can include a process and an end state. Focusing on consumers’ individual experiences of CGHD and engagement as an end state, “outcome” is consistent with the patient centric aspirations of this study. Hence, this research explores an ontological engagement framework of cognitive, emotional, behavioral, and physiological outcomes for the use of consumer data to answer the research questions: *How are consumer generated health data used by consumers? How does this data influence engagement?*

## Methods

Study 1 was conducted to explore experiences of CGHD, whereas study 2 was used to validate these findings in clinical care and explore carer-generated data. This 2-study research agenda improves construct validity through cumulative validation and confirmation by key informants [22,23] and clinician interviews used to validate consumer perspectives.

Study 1 sought a purposive sample of patients, carers, and doctors who were experienced in consumer-generated photography and used semistructured interviews to explore data use. Maximum variation sampling was applied by sampling from across the medical subspecialities of general practice, emergency health care, surgical care, and trainee. In addition, all consumers were judged as once-off users, intermittent users, regular users, and constant users of CGHD. This offered an opportunity to explore the widest possible attitudes, perceptions, and beliefs about the data via a cohort who understands the implications and complexity of its use. The sample size used in qualitative research relies on idea saturation to ensure reliability, and 30 participants was deemed suitable.

Study 2 utilized a pilot clinical trial with theoretical sampling of 30 parents of children undergoing laparoscopic appendectomy surgery at a large tertiary children’s hospital. Using a standard operating procedure, parents were trained to take photographs of their children’s surgical site wounds every 2 days and email them to the staff for the 10 days after the procedure. An all-comers approach consistent with other laparoscopic interventions was used [24]. Postintervention interviews were conducted by the researcher using introductory open and probing questions to deepen the understanding of CGHD use, engagement, and to validate the ontological framework.

Using grounded theory, interviews were analyzed using constant comparison for meaning using a 5-step process: (1) Gaining familiarity through reading all transcripts, (2) Data reduction via coding, (3) Thematic analysis of the codes, (4) Data reorganization into the 4 domains and comparison across both studies, and (5) A discussion of the key considerations related to the research question [25].

The 2 studies were analyzed sequentially but iteratively compared. Codes and themes from study 1 (perceptions of the CGHD) emerged from the data, then were then mapped to the ontological framework. Study 2 (use of data in care) provided validation of the existing codes and themes and contributed new information. When new codes and themes emerged from study 2, they were compared and validated by study 1 findings and reorganized into the final framework. The combination of the 2 approaches ensured consumers’ perceptions of CGHD use matched actual use in clinical care.

## Results

### Overview

Saturation was achieved at 60 interviews. In study 1, 19 males and 16 females participated in 34 interviews (Multimedia Appendix 1). Participants were aged between 22 to 69 years. A total of 16 patients and 7 carers were from a wide range of socioeconomic situations, with doctors from the 4 predefined categories. In study 2, 5 male and 21 female carers completed the trial by taking between 1 and 6 photographs of their child’s surgical site wounds over 10 days (Multimedia Appendix 2). Of the original 30, 4 participants did not complete the trial. Participants were aged between 18 to 70 years, with children aged between 1 to 17 years.

Although there was significant similarity, study 1 and study 2 contributed unique findings to the results. Study 1 provided the 8 themes of improved health outcomes, self-perception, emotional regulation, empowerment preventative mind-set, self-management, social support, and partnership with providers, with study 2 contributing service optimization and service assessment. Although there were no major differences between patients and carers, doctors contributed the finding of patient deviance, which was supported then by carers.

In this context, CGHD has 30 main use outcomes grouped into 10 themes fitting within the ontological framework. Although outcomes have been placed into the physiological, cognitive, emotional, and behavioral groups, engagement is contextual and dynamically present in individual consumers. Outcomes were thus found to overlap and be interrelated, consistent with previous seminal research [21]. When the health care consumer perspective is analyzed, the physiological, cognitive, emotional, and behavioral outcomes of engagement are present but with a strong emphasis on the emotional outcomes of CGHD-related engagement.

### Improved Health Outcomes

If you can take photos over time of visual things like rashes and growths and whatever, you can see whether they’re getting worse or better. It’s easy to miss subtle changes over time.

Female Patient

I also do think that there is a certainly much bigger potential in terms of research, and you know, into ongoing treatment or into the monitoring all sorts of illnesses.

Male Carer

Data had multiple, overlapping uses for physiological engagement including diagnosis, management, and medical research. Diagnosis and management of transient conditions, chronic conditions, slow healing, and slow-progressing conditions occurred with CGHD. This was enabled through the recording of changes and treatment over time. Doctors’ opinions of the data were mixed, with it considered redundant information, partial information, and key information. A specific area of both opportunity and concern was remote diagnosis. Patients and carers agreed that remote diagnosis was possible with these data, indicating it overcame barriers of distance improving their quality of life, saving them time and money [4], although clinicians were skeptical about diagnosing without seeing the patient in person. In addition, CGHD can be used for research purposes, with the majority of discussion about projects led by health care staff. Patients were comfortable sharing CGHD, providing adequate consent is obtained. The caveat to this was that patient age, sensitivity of the condition, sensitivity of the body part, and anonymity were all factors in the consent process.

### Self-Perception

With the breast cancer, the motivation [to take images] was this weird thing. I had to remember what I looked like before the surgery and come to terms with the fact that it’s really happening to me. It’s like a concrete object that reflects what’s happening.

Female Patient

I think it [photography] makes me feel like a more responsible by engaging in that process...It definitely made me feel like you've been more responsible about the healing of the wound, without a doubt.

Female Carer

One of the most discussed themes in all interviews was the notion the data were evidence of a patient’s personal record and of the medical experience. Recording the health care journey is a preliminary step toward health care sense-making and a reaction to the stressors of disease, which is intended to generate a sense of coherence [26]. In short, generating data is an expression of a patient’s desire for control in the face of a stressful illness and helped consumers make sense of what was occurring. Patients documented their bodies before and after medical interventions as an act of both vanity and the documentation of survivorship [27]. Carers suggested the data were going to kept as mementos for adolescent children so that when they grow up they have a record about what they have gone through in their own lifetime.

Responsibility was a category that emerged as important for both patients and carers when discussing CGHD. Taking on extra responsibility through documentation was considered a consequence of data use. Increasing a patient’s responsibility was suspected to improve health outcomes; however, doctors were wary with many concerned about this practice. One commented:

The disadvantage is that – I guess, the patient is now responsible for looking after the moles themselves, they may slightly be falsely assured in some way.

Emergency Medicine Consultant

### Service Assessment

The opportunity to send those pictures and assess the risk of infection and things like that to a trained professional have a look like rather than making your own judgment or Googling. I think that’s really important, and that’s what I mean when I say satisfaction.

Male Patient

If that [remote photography assessment] became a regular thing I would have confidence in the system rather than the individual doctors. I would have more confidence that the problem has a chance of being resolved...none of the doctors are infallible.

Male Carer

Trust for the doctor, satisfaction with the service, and service confidence were all considered cognitive outcomes of the use of CGHD in clinical care; however, consumers did acknowledge trust was influenced by more than just using the data. Indeed, patients thought using the data may signal an increase in their trustworthiness and equate to more respect from the clinician in the service interaction. Doctors agreed that using data increased a sense of trust:

Patients feel that we trust them...it sort of creates a relationship where the patient is sure the doctor trusts this picture.

Surgical Registrar

In general, service assessment relies on evaluating the frontline service interactions through a prism of relational factors between the consumer and provider [28]. Satisfaction was present and discussed in interviews but remains an indistinct concept, conflated with service confidence, reassurance, and going beyond what is expected. Study 1 suggested CGHD could improve satisfaction, and in study 2, many parents commented that was indeed the case. One female carer was enthusiastic about her service experience, commenting that satisfaction was about “[when] those photos had gone back to the surgeon you know that that feels like going above and beyond the normal standard service.”

### Preventative Mind-Set

I was probably just made more aware of just healthcare in general by being involved in taking the photos.

Female Carer

They [wound photographs] just reminds me that I’ve got to watch what I’m doing. As the doctor explained to me when I was leaving the hospital, he says because I’ve got diabetic feet which is neuropathy in the feet, I’ve gotta’ wear shoes all the time.

Male Patient

CGHD increased awareness of a condition and promoted health behaviors. This was most clearly expressed by 1 patient who noted photographs reminded her to reduce her stress levels to improve facial acne. Although it is clear that data can help awareness and serve as a reminder for healthy behaviors, recent literature suggests that although engagement using devices has the potential to facilitate health behavior change, this might not be driven by devices alone [29]. Commensurate with these findings, this research suggests data were a facilitator and prompt for behaviors, not a mechanism for change in itself.

In study 2, parents who documented their child’s wounds used data collection as an excuse and reminder to check healing progress. It was discussed that adolescent children may not allow parents to observe wound healing, but the photography was “for the doctor” and therefore permitted. Interestingly, participants used the photography as a prompt to self-manage care episodes, with 1 parent saying:

It was really good for me because otherwise my management of time can be quite poor sometimes and I would - may forget to - I probably would have forgotten to check his wound.

Female Parent

### Emotional Regulation

I did that [taking photographs] around the time I started caring for Dad. You start noticing all these other things when you start caring for someone, so it also worked well for my own peace of mind. It's reassurance, yes they will believe that yes this has occurred, and this is ongoing.

Female Carer

I was going to take a photo of my butt because I had a big bruise there, but that wasn’t for any, it wasn’t for any kind of diagnosis or anything. That was just posting on social media because, you know, getting a response from people.

Male Patient

CGHD provided reassurance by helping consumers cope with difficult emotions, and this phenomenon was recognized by all 3 participant groups. Patients and carers used data for increased emotional reassurance that healing was occurring and that they were performing the required tasks to get better. In opposition to reassurance, anxiety was suggested as creating data might cause undue stress. Interestingly, both doctors and patients recommended that capturing data may increase the anxiety related to the condition, and hence, not all participants would be suitable for data collection. Indeed, although data collection may be a sign of diligence in a patient, it might also be a sign of hypochondria, tracking conditions that are not required, which in turn cause distress.

CGHD offered consumers an avenue to share data with others as a form of entertainment within the peer network and to improve the lives of others through altruistic acts. Despite being characterized as “grotesque” and “embarrassing,” male participants used the graphic nature of medical images for play and entertainment of others. Patients were also motivated to share data by a feeling of altruism, giving information to benefit the wider community, which is commensurate with Spencer et al [30] who reported 98% of participants who shared anonymous health information for research purposes “considered that the altruistic benefits of sharing health care data outweighed the risks.”

### Empowerment

[With videos] the doctors can see that I’m not making this thing up. I had a history of depression before so they would say, “well, funny things can happen when you get depressed.” So, it was valuable for me to be able to show it to someone, like “here it is”… I became more of an advocate myself.

Male Patient

When the patient takes the photograph, the patient themselves has thought, “Oh this is something. I need to do something about it and actually pick up the camera.” To do that gives you a little bit of sense of control.

Female Carer

The most important finding of emotional engagement is that generating data was an empowering process promoting self-advocacy, self-confidence, and a feeling of control. A patient with a history of depression suffered intermittent hand twitching generated medical videos for a diagnosis. As such, the data provided an avenue for self-advocacy, giving the consumer more credibility with health care professionals. Self-advocacy was also present when carers used data to assert their role within families. Data focused conversations with family, making the process of healing easier and reassuring the carers that patients were improving. Data also promoted self-confidence, suggesting they were functioning as a “good” carer.

Furthermore, establishing a sense of control was important to many consumers, who felt health care services can be “dehumanizing” and “disempowering.” Generally, the sense of control was expressed as both health situation and health system control. CGHD use improved the patient’s feeling of equality with the health care provider and helped access health care services in a timely manner equating to a sense of control over the health care service. At home, it reassured patients and carers that they had a role in managing care, improving their perception of control over the health situation.

### Self-Management

The main thing I wanted to track was the rate at which it was healing. So, I used to take photographs, along with a ruler on the side of the wound and that way, we could actually pick up, at some point that the wound had arrested.

Male Patient

If you wanted to do some self-diagnosis, you've got the proof sitting there behind you of these photos.

Female Parent

Even before digital devices, patients were offered opportunities to monitor their own condition. This occurred for chronic diseases such as diabetes and improved self-efficacy, even in patients from diverse backgrounds [31]. It was universally observed by patients, carers, and doctors that digitally created CGHD is used for self-monitoring and self-education, which can lead to the self-diagnosis. Patients considered the use of Dr Google a health care right but realized that not all information is accessible or reliable. Finding a legitimate source for self-education was considered difficult because of multiple illegitimate sources, and the readability of most authoritative Web pages exceeds average national levels of literacy [32].

Doctors stressed that although the data may be useful for self-diagnosis, they can lead to misdiagnosis and misinformation. Misinformation has medico-legal implications for health care professionals, and although 1 doctor commented they would never be “stupid enough” to diagnose from consumer-generated data, others acquiesced, suggesting that treating the patient also means treating data and the ideas they present with. Refusal to engage with patients who present data will impact the service experience. Indeed, patients who present data-evidenced opinions switched doctors if their perspectives were not recognized.

### Social Support

I think it’s a bit of a connection thing. If people do see it [a photograph] and they say “Oh,” you know, “I can sympathise,” and I think that’s what the medical profession is lacking.

Male Patient

I have a colleague at work who is also a very good friend. She's having surgery on Monday I was glad I had my daughter’s photos and was able to send my friend at work the progressive photos to show her how well the wounds were healing.

Male Carer

Data has an important function in the social support of patients with both data sharing and information seeking, improving a sense of connectedness through internet surrogates. Data sharing in online and offline networks for social support was very common. For example, an older female patient was supported through her knee reconstruction and encouraged to continue treatment with supportive comments that she was “doing well” and to “keep up the good work.” This is concordant with literature that suggests social networks support adherence to short duration activity regimes [33].

CGHD was shared with the patient network for an update on the patient status and information seeking in study 1. Notably, no information seeking was observed in study 2, disconfirming the use of the data in this context. When participants were asked why, approximately half mentioned the absence of this behavior, suggesting they did not need to search online or use the peer network for information seeking, potentially reducing instances of self-diagnosis. The reasons given were both that a diagnosis was already made, and that because of the remote support of the doctors’, self-diagnosis was not required.

### Partnership With Providers

By the time I saw my doctor I had a skin graft taken and applied to my arm, so it looked pretty normal. However, the photos after the operation, when I was changing the dressing, pretty much showed an arm that was cut from elbows to palm with all the muscles sort of hanging out, sitting on the table. And that it would have been impossible for the doctor to understand or see that scenario without taking photos.

Male Patient

I took a photo and in a series of about 3 hours, you could see the breakdown of it [the wound] happening; we were able to use it as a thing, to say this needs urgent attention now…I bumped into one of the doctors and she told the surgeon…I think I showed her the photo of it breaking down and she called the surgeon, and the surgeon stepped in.

Female Carer

CGHD instigated cooperation between consumers and providers, improved communication, and gave the provider an opportunity to offer parents support by distance and equated to more perceived respect for the consumer. Improved patient–provider communication was a well-documented outcome of data use [34]. It was universally discussed by patients, carers, and doctors that a feature of photographic data was that it overcame an inability to describe a condition. Furthermore, when the data were used in clinical care, it could be used as a focus of conversation or for agenda setting. It prompted questions and feedback with participants commenting the data improved meeting participation with health care providers. Importantly, the communication was bidirectional, with clinicians also experiencing the benefit of improved communication, suggesting it prompted other questions they might not have asked.

In addition to improved communication, cooperation was noted in interviews. Data were used to educate and motivate clinicians, increasing a sense of urgency for the treatment of patient conditions. Images were not only used as a “call to action” between the patient and doctor, but clinicians used patient data to convince their colleagues to urgently treat their patients. Consumers understood the “power relationship in a medical model,” however, they sought cooperation with clinicians when using CGHD. It was suggested that health care professionals could cooperate with patients by training them on the data capture, and clinicians agreed it was part of their job to educate patients. If cooperation was not enacted, for example, by “shrugging off” consumer data, it caused dissatisfaction with the service experience.

Consumer-generated data affected parents’ perceptions of the service through supported autonomy at home. Patients and carers experienced a sense of autonomy in their health situation, however, are still supported by health care professionals within the health system through remote diagnosis of the images. This helped rebuke the feeling of “having the door swing shut on you once you have left the service” and was considered going above and beyond what was expected, extending the service relationship beyond the clinical context.

Finally, a regular theme prominent in study 2 was parents recognizing that through the act of providing data, they gained greater attention and more respect. Typically, this was expressed as being taken more seriously. Participants attributed this to being more aware of the health condition, proactive when caring, and taking on greater responsibility for their child’s health. A total of 1 patient experienced greater respect when she alerted medical staff to an infection discovered through consumer-generated photography:

I think that they probably may take you a little bit more seriously because you said you were aware that you know 12 hours ago that wasn't there, or six hours ago that wasn't there, and then suddenly it was something like we have maybe a bit more respect for parents.

Female Parent

### Service Optimization

You can monitor things yourself. If you notice a significant change in 6 months’ time, because you’re looking at yourself and you can compare it with the original photo that was taken. So, you might go back to the doctor in 6 months, rather than waiting the 12 months for your scheduled check-up.

Male Patient

[Using photographs] people might have demanded to see a surgeon straight away, or, whatever and I probably just tried to stay calm, and just say, you know, “when you got to me that you just popped in here and have a look at this.”

Female Carer

Patients and carers experienced a greater ability to effectively manage their own resources and reduce health care services use by eliminating unnecessary appointments. The main way patients used data in this context was to alter their own treatment plan, but this could also lead to deviant behaviors. Typically, this meant reviewing photographs for evidence of clinical improvement and then making appointment changes based on symptoms shown in the data. Consumers commented the data could provide detection of adverse health events, motivating an early visit to a health care service. Doctors, patients, and carers all agreed that getting timely appointments for transient conditions was difficult and that health services lacked continuity between appointments, with CGHD overcoming these limitations. Finally, when clinician’s expertise was limited, CGHD could be sent to experts for review.

Deviant customer behavior refers to actions that patients take to abuse the health care system that violate accepted norms of behavior and result in harm [35]. The research found no evidence that patients will take actions that affect other patients but may exhibit deviant behaviors toward the health care provider. Carers and doctors, but not patients, agreed that deviant behavior involved asking for diagnosis based on the data for someone not present; self-diagnosis, which delays seeking medical help; and insisting that the patient’s interpretation of the data is correct despite contradicting the clinical expert. A total of 4 doctors commented that when patients use the data for self-diagnosis, they often come to the consultation with a preconception about what they have. When diagnosis is inconsistent with expectations, patients may get a second opinion and change providers.

**Figure 1:**
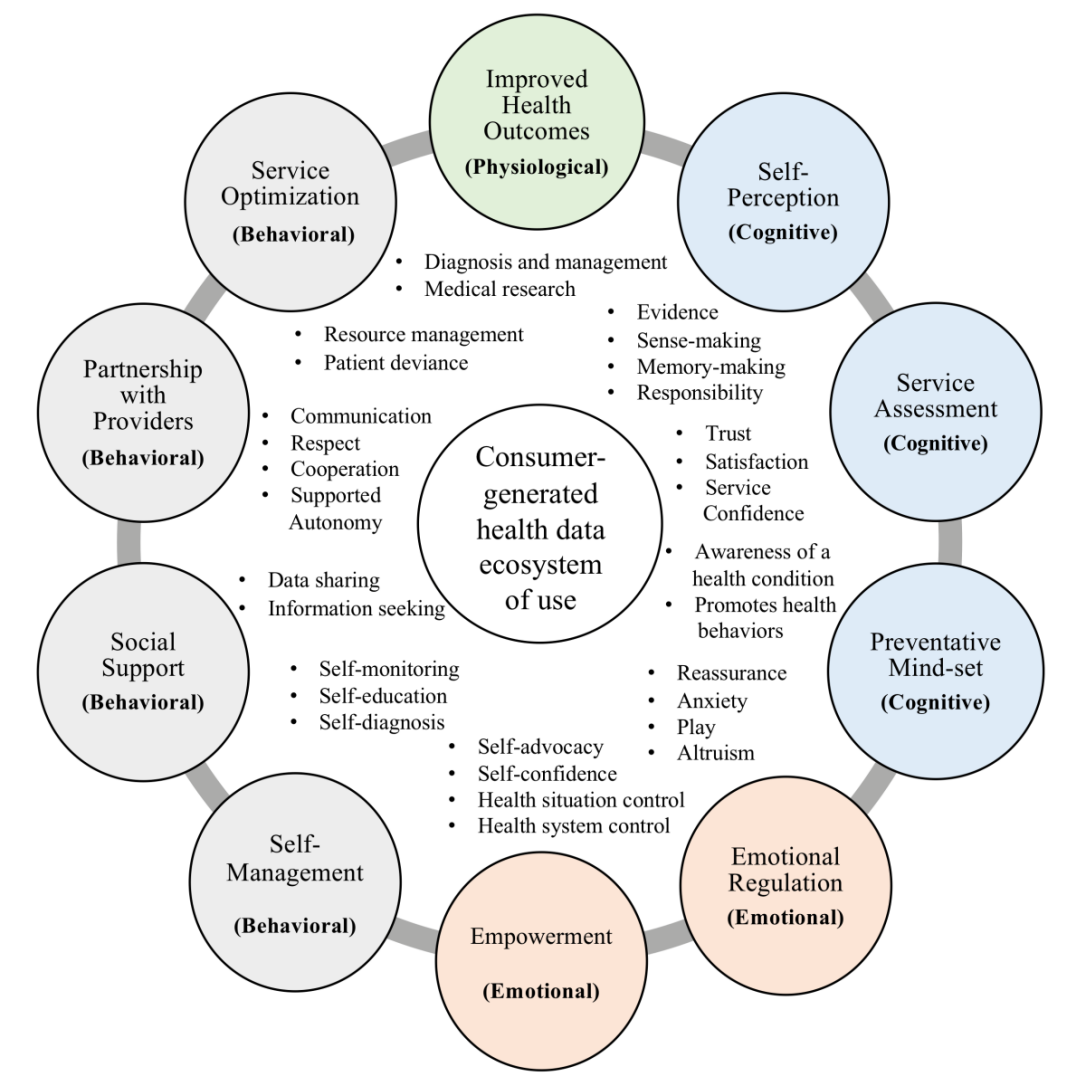
An engagement framework for consumer-generated health data.

As clinical costs rise, private telehealth offerings emerge, and health tracking gains popularity. Providers face unprecedented pressure to develop cheaper, patient-centric, value-based health services. Engaging patients has become a key focus patient-centered care and has the potential to improve both the quality and efficiency of health care services. CGHD can improve service accessibility and promote engagement. Thus, Figure 1 demonstrates CGHD use attributes go beyond the physiological gains of improved health outcomes to incorporate an ecosystem of physiological, cognitive, emotional, and behavioral engagements (also see Multimedia Appendix 3).

## Discussion

### Principal Findings

Having those medical photos going to a medical practitioner has meant that I felt more and more engaged, more involved and more responsible. I was given some sort of capability to assist in the process.

Female Carer

Consumers use data for improved health outcomes, self-perception, service assessment, emotional regulation, empowerment, social support, partnership with providers, and service optimization. For providers, data can aid diagnosis and management, improve communication, and can reduce unnecessary consultations. CGHD is another tool to manage the patient journey, allowing providers an opportunity to assure consumers that healing is occurring. For consumers, data aides sense making, instigates greater personal responsibility for health care outcomes, can foster improved awareness of a condition, and promotes healthy behaviors.

### Limitations

The concepts demonstrated have been explored qualitatively, and no quantitative confirmation of relationships was undertaken. In addition, many of the themes and uses overlap, and some may be categorized into more than 1 area. The subjective nature of qualitative research does introduce the possibility of researcher bias; however, the techniques of intercoder reliability, maximum variation sampling, and a theoretical framework developed from the patient engagement literature were used to mitigate these issues. A further limitation is that patients may have very different perspectives on CGHD use, with other technologies and clinical scenarios thus impacting on engagement.

### Comparison With Prior Work

Although patients and carers wholeheartedly supported data use, 1 clinician refused to see the data at all. This confirms other findings that a small percentage of health care practitioners will not be interested CGHD and may miss the opportunity to improve the therapeutic relationship [3,36]. Importantly, this study confirms and explains why empowerment occurs, suggesting it is an experience of self-advocacy, self-confidence, health system control, and health situation control and more than just “being part of the team” [8].

### Conclusions

Today, consumers are connecting in online and offline networks, collating health information through mobile technology, and looking for innovative services that improve their participation. Thus, managers should aim to develop strategies to promote CGHD, as refusal to engage with patients who present data will impact service experience. Indeed, our research shows patients who present data-evidenced opinions will switch doctors if their autonomy is not recognized.

Furthermore, patients are much more likely to attempt to self-diagnose using consumer-generated data when the doctor was not available and when they have a peer network. Therefore, “prescribing” data collection may reduce instances of self-diagnosis. If data are supported then it can lead to higher service satisfaction, higher service confidence, and an improved therapeutic relationship. Finally, future research should address the use of other data in other clinical settings adding to the existing theoretical framework limited to acute and chronic health care.

## Appendix

Multimedia Appendix 1 Study 1 participant characteristics.

Multimedia Appendix 2 Study 2 participant characteristics.

Multimedia Appendix 3 Themes and participant quotes.

## Abbreviations

CGHD: consumer-generated health data
