# Supplementary material for: Creating Consumer-Generated Health Data: Interviews and a Pilot Trial Exploring How and Why Patients Engage
Source: J Med Internet Res. 2019 Jun 13;21(6):e12367. doi: 10.2196/12367 (PMC6598415; doi:10.2196/12367)
Supplement: Multimedia Appendix 2 [file jmir_v21i6e12367_app2.docx]

**Appendix 2:** Study 2 Participant Characteristics

| **Enrolled** | **Participant ID** | **Gender** | **Parent Age (yrs)** | **Childs Age (yrs)** | **Education** | **Income** | **Rurality** | **Interview** |
| --- | --- | --- | --- | --- | --- | --- | --- | --- |
| 1 | Par01_F_Rural | F | 31-40 | 4-12 | Degree | >$120,000 | Rural | Yes |
| 2 | Par02_F_Urban | F | 41-50 | 4-12 | Degree | >$120,000 | Urban | Yes |
| 3 | Par03_M_Urban | M | 41-50 | 4-12 | Degree | $80,001-120,000 | Urban | Yes |
| 4 | Par04_F_Urban | F | 51-60 | 12 + | Degree | >$120,000 | Urban | Yes |
| 5 | Par05_F_Urban | F | 31-40 | 1-4 | Degree | $80,001-120,000 | Urban | Yes |
| 6 | Par06_F_Urban | F | 41-50 | 4-12 | Degree | >$120,000 | Urban | Yes |
| 7 | Par07_F_Rural | F | 31-40 | 4-12 | TAFE | $80,001-120,000 | Rural | Yes |
| 8 | Par08_F_Rural | F | 41-50 | 4-12 | TAFE | $30,001-50,000 | Rural | Yes |
| 9 | Par09_M_Urban | M | 61-70 | 12 + | Post Graduate | >$120,000 | Urban | Yes |
| 10 | Par10_F_Urban | F | 41-50 | 12 + | Post Graduate | $80,001-120,000 | Urban | Yes |
| 11 | Par11_F_Urban | F | 31-40 | 1-4 | High School | $50,001-80,000 | Urban | Yes |
| 12 | Par12_F_Urban | F | 41-50 | 12 + | Degree | >$120,000 | Urban | Yes |
| 13 | Par13_M_Rural | M | 41-50 | 4-12 | High School | $80,000-120,000 | Rural | Yes |
| 14 | Par14_F_Rural | F | 31-40 | 4-12 | High School | $80,000-120,000 | Rural | No |
| 15 | Par15_F_ Rural | F | 41-50 | 12 + | Degree | $80,000-120,000 | Rural | Yes |
| 16 | Par16_F_Urban | F | 31-40 | 4-12 | Post Graduate | $80,000-120,000 | Urban | Yes |
| 17 | Par17_F_Urban | F | 41-50 | 4-12 | Degree | $50,001-80,000 | Urban | Yes |
| 18 | Par18_M_Urban | M | 51-60 | 4-12 | TAFE | >$120,000 | Urban | Yes |
| 19 | Par19_F_Rural | F | 18-30 | 1-4 | TAFE | <$30,000 | Rural | Yes |
| 20 | Par20_F_Urban | F | 31-40 | 4-12 | Degree | $50,001-80,000 | Urban | No |
| 21 | Par21_F_Urban | F | 41-50 | 12 + | Degree | $30,001-50,000 | Urban | Yes |
| 22 | Par22_F_Urban | F | 31-40 | 4-12 | Post Graduate | >$120,000 | Urban | No |
| 23 | Par23_F_Urban | F | 31-40 | 4-12 | High School | $50,001-80,000 | Urban | Yes |
| 24 | Par24_F_Urban | F | 18-30 | 1-4 | High School | $50,001-80,000 | Urban | No |
| 25 | Par25_F_Urban | F | 41-50 | 4-12 | Degree | $80,001-120,000 | Urban | Yes |
| 26 | Par26_F_Urban | F | 41-50 | 4-12 | Degree | $30,001-50,000 | Urban | Yes |
| 27 | Par27_M_Urban | M | 41-50 | 12 + | Degree | $80,001-120,000 | Urban | Yes |
| 28 | Par28_F_Urban | F | 51-60 | 4-12 | High School | $30,001-50,000 | Urban | Yes |
| 29 | Par28_F_Urban | F | 31-40 | 4-12 | Degree | $30,001-50,000 | Urban | Yes |
| 30 | Par30_F_Urban | F | 41-50 | 4-12 | Degree | $30,001-50,000 | Urban | Yes |
