# Supplementary material for: Creating Consumer-Generated Health Data: Interviews and a Pilot Trial Exploring How and Why Patients Engage
Source: J Med Internet Res. 2019 Jun 13;21(6):e12367. doi: 10.2196/12367 (PMC6598415; doi:10.2196/12367)
Supplement: Multimedia Appendix 3 [file jmir_v21i6e12367_app3.docx]

**Appendix 3:** Themes and participant quotes

| **Theme: Improved Health Outcomes (Physiological)** | |
| --- | --- |
| Diagnosis and management | **S1** “*I think the photographs would be useful, obviously combined with the consultation because skin rashes can be so many, and varied, and look alike.”* Pat08_MF54_R |
|  | **S2** “*[The photos were used] to see the healing in my child after surgery, post op, after the operation”* Par05_F_Urban |
| Medical research | **S1** *“I also do think that there is a certainly much bigger potential in terms of research and you know, into ongoing treatment or into the monitoring all sorts of illnesses.”* Car05_M56_R |
|  | **S2 “***What you're doing [data collection] is valuable to them [doctors], for their research for their own medical studies and for surgeons in the future”* Par08_F_Rural |
| **Theme: Self-Perception (Cognitive)** | |
| Evidence | **S1** *“it’s a record of time more than anything else…all that stuff’s visual journal, what’s been going on, and you can always relate that to later on as well*.” Pat01_M41_O |
|  | **S2** *So therefore you're still dealing with a sick child, but there is something about it that made me feel that I had some sort of evidence of her getting a bit better each day that there was some sort of reference that I could use if by any chance it looked like she was getting worse.* Par04_F_Urban |
| Sense-making | **S1** *“with the breast cancer, the motivation [to take images] was this weird thing. I had to remember what I looked like before the surgery and come to terms with the fact that it’s really happening to me. It’s like a concrete object that reflects what’s happening.*” Pat08_MF54_R |
|  | **S2** *“I just wanted to see the area or the progress or was it going to get worse. I was going to find out whether there was any infection I think last time. If there was an infection, I think I could figure out there was going to be some white things or swollen or that sort of stuff.”* Par27_M_Urban |
| Memory-making | **S1** “*I’m kind of thinking about doing something kind of arty with them in some way, making a piece of artwork about them. And it will sort of be like something that I’ll go back to and it will bring back memories of the process.”* Pat08_MF54_R |
|  | **S2 “***You know when they grow up [children], to have our own record about what they have gone through in their own lifetime. And that’s what happened to her appendix and it is good to have it as a family record.”* Par05_F_Urban |
| Responsibility | **S1** *“I mean, with a thing like this [photography]… when you’re doing your own dressing changes, and things like that, you feel a much, much greater responsibility in terms of your own care.”* Pat02_M48_R |
|  | **S2 “***by having to sort of do the task you could have just look at the scars and say “I don't really think there's really a problem with this scars”. I think it makes me feel like a more responsible by engaging in that process. Yeah, definitely made me feel like you've been more responsible about the healing of the wound, without a doubt.* Par03_M_Urban |
| **Theme: Preventative Mind-set (Cognitive)** | |
| Awareness of a health condition | **S1** *“They [wound photographs] just reminds me that I've got to watch what I'm doing. As the doctor explained to me when I was leaving the hospital, he says because I've got diabetic feet which is neuropathy in the feet, I’ve gotta’ wear shoes all the time.”* Pat14_F62_R |
|  | **S2** *“I was probably just made more aware of just healthcare in general by being involved in taking the photos.”* Par16_F_Urban |
| Goal setting | **S1** *“Yeah, it [photographs of my face] reminded me that’s I should not go too hard sometimes. I should relax to release my stress otherwise, I will not have more pimples on my face. Sometimes it’s a reminder to remind you to do more exercise.”*Pat15_F30_I |
|  | **S2** *“It was really good for me because otherwise my management of time can be quite poor sometimes and I would - may forget to - I probably would have forgotten to check his wound.”* Par06_F_Urban |
| **Theme: Service Assessment (Cognitive)** | |
| Trust | **S1** “*I think if you’ve got a good doctor, and I know not everyone has…you build a relationship …there is a level of trust there that says I can bring you this information…I can bring the information and you’ll accept it.”* Pat04_F38_I |
|  | **S2** “*I think I have more trust in the systems that are in place to make sure those things get done.”* Par03_M_Urban |
| Satisfaction | **S1** *“I don't know if that’s offered at other hospitals [name removed] with extra care afterwards and things like that and the opportunity to send those pictures and assess the risk of infection and things like that to a trained professional to have a look like rather than making your own judgment or Googling. I think that’s really important, and that’s what I mean when I say satisfaction.”* Pat03_M33_I |
|  | **S2** *“[when] those photos had gone back to the surgeon you know that that feels like going above and beyond the normal standard service.”* Par10_F_Urban |
| Service Confidence | **S1** Absent |
|  | **S2** *“If that [remote photography assessment] became a regular thing I would have confidence in the system rather than the individual doctors.* *I would have more confidence that the problem has a chance of being resolved…none of the doctors are infallible.”* Par03_M_Urban |
| **Theme: Emotional Regulation (Emotional)** | |
| Reassurance | **S1** *“It [sharing photos] was pretty positive, actually. I got some comments ‘yeah mines worse than that’ on why I was doing that, but I got some comments saying, ‘yeah, mine started out like that’, and giving a particular kind of reason – You know, your fingers just shouldn’t move by themselves without you wanting to grab a glass or grab a pen or something, and it was a pretty weird. I guess I got some reassurance from people online.”* Pat10_M49_I |
|  | **S2** *“I would have thought to look at it for two days and if it's not sweating or whatever you don't worry about it. But there's no way to sort of checked it 10 days later…I think there's a reassurance in that.”* Par03_M_Urban |
| Increased Anxiety | **S1** *“if he or she is going to share the photo with the GP, it is better not to share it before with the family or any, or a social media… as a GP, I prefer to be the first one to be consulted for that, you know, medical condition before, because usually people before coming to the GP may get non-informative feedback from others, from friends, from family members. So, these kinds of feedback may increase their, you know, anxiety.”* Pat14_F62_R |
|  | **S2** *“It can get a bit troubling I guess it is, if he thought there was something wrong with the wound, and the people viewing them were saying it was all right. That’s time it might make you feel less reassured.”* Par03_M_Urban |
| Play | **S1** “*I mean, the other day, I was going to take a photo of my butt because I had a big bruise there, but that wasn’t for any, it wasn’t for any kind of diagnosis or anything. That was just posting on social media because, you know, getting a response from people.”* Pat01_M41_O |
|  | **S2** *“I think they [my children] don't quite like it [the wound photo], it looks a bit disgusting. …I think we made it a bit fun, you know…it's disgusting.* Par27_M_Urban |
| Altruism | **S1** *“Obviously, for the care of the patient foremost, but I mean I don't, I'm not opposed to them [photographs] being used for training purposes, or research purposes and that sort of thing to the benefit of mankind.”* Car04_M45_1 |
|  | **S2** *“they might find something [in the images] that might actually then help better the process for someone else.”* Par23_F_Urban |
| **Theme: Empowerment (Emotional)** | |
| Self-advocacy | **S1** “*the doctors can see that I’m not making this thing up. I had a history of depression before so they would say, ‘well, funny things can happen when you get depressed’. So, it was valuable for me to be able to show it to someone, like ‘here it is’… I became more of an advocate myself.”* Pat10_M49_I |
|  | **S2** *“If I had a question or if there was something that wasn't - or whatever - I felt okay to talk about it and the staff were listening.”* Par16_F_Urban |
| Self-confidence | **S1** *“sometimes I take a photo and look away and take a photo. But my husband’s can be confident that yes, it is healing and it is improving. So it gives him confidence to be able to self-monitor which is a big thing when you’ve been very ill.”* Car01_F56_R |
|  | **S2** *“Just more confident with actually taking the photos if required, and in general in the, the appearance of the wound and recovery I suppose.”* Par16_F_Urban |
| Health System Control | **S1** *“it [the photography] all turned into taking control of it yourself, probably because of the fact that I went into hospital a year prior to the accident with pneumonia and I was getting tossed about from specialist to GP to this, that and the other and no one seemed to be sort of taking initiative of the entire situation.”* Pat07_M56_R |
|  | **S2** *“I think you feel a little bit empowered that you're not getting a response from someone who had the scar to say that it's so terrible.”* Par03_M_Urban |
| Health Situation Control | **S1** *“when the patient takes the photograph, the patient themselves has thought, “Oh this is something. I need to do something about it and actually pick up the camera.” Uhm to do that gives you a little bit of sense of control.”* Par05_F_Urban |
|  | **S2** *“1 felt some control because it allowed me to think about thing of being able to a teenage child be able to communicate with them…having this as a kind of interaction, it really helped us.”* Par04_F_Urban |
| **Theme: Self-Management of a Health Condition (Behavioural)** | |
| Self-monitoring | **S1** *“The main thing I wanted to track was the rate at which it was healing. So, I used to take photographs, along with a ruler on the side of the wound and that way, we could actually pick up, at some point that the wound had arrested.”* Pat02_M48_R |
|  | **S2** *“Taking the photos I was able to flick back to the previous photos and look at how it was changing.”* Par02_F_Urban |
| Self-educating | **S1** *“People have a right to research and look up Dr. Google…but at the end of the day…I think it’s the doctor’s job and profession, at the end of the day, to actually apply their expertise and make a diagnosis and treatments.”* Pat04_F38_I |
|  | **S2** *“It's just reassurance that you know that they're at that right stage if either were, you know, or online researching to see if they're at the right stage.”* Par07_F_Rural |
| Self-diagnosing | **S1** *“The internet has a million websites these days, that self-diagnose for people without seeing doctors.”* Pat04_F38_I |
|  | **S2** *“If you wanted to do some self-diagnosis, you've got the proof sitting there behind you of these photos.”* Par07_F_Rural |
| **Theme: Social Support (Behavioural)** | |
| Data Sharing | **S1** *“My husband had his skin cancer cut off… and some of them were quite ugly… and so we have used it to prepare the grandchildren for changes in, that this is why grandpa is so sick, that this is why grandpa is so sore. I don’t know if it’s education, but just in explaining the procedures to the younger members of the family. Because it’s less yucky [seeing a photo] than actually seeing it as a big skin graft on his hands.”* Car01_F56_R |
|  | **S2** *“I have a colleague at work who is also a very good friend. She's having surgery on Monday I was glad I had my daughter’s photos and was able to send my friend at work the progressive photos to show her how well the wounds were healing.”* Par11_F_Urban |
| Information Seeking | **S1** *“with social media, I think it’s a bit of a connection thing. If people do see it [a photograph] and they say ‘Oh,’ you know, ‘I can sympathise’, and I think that’s what the medical profession is lacking. Like when you go and see a family doctor in the past, they give you some kind of comfort with their words and that kind of stuff.* Pat01_M41_O |
|  | **S2 Absent** |
| **Theme: Partnership with Providers (Behavioural)** | |
| Communication | **S1** *“By the time I saw my doctor I had a skin graft taken and applied to my arm, so it looked pretty normal. When I say normal, there was still stitching from my palm to my elbow. However, the photos after the operation, when I was changing the dressing, pretty much showed an arm that was cut from elbows to palm with all the muscles sort of hanging out, sitting on the table. And that it would have been impossible for the doctor to understand or see that scenario without taking photos.”* Pat07_M56_R |
|  | **S2** *“When things go wrong it is usually over a weekend or in the evening when you can’t go to a doctor, I think definitely that if you could use that for communication.”* Par02_F_Urban |
| Respect | **S1** *“I was able to monitor my own progress, I was treated with a certain amount of respect that I had the intelligence to sort of keep an eye on this.”* Car05_M56_R |
|  | ***S2*** *“I think that they probably may take you a little bit more seriously because you said you were aware that you know 12 hours ago that wasn't there, or six hours ago that wasn't there, and then suddenly it was something like we have maybe a bit more respect for parents that are a bit more aware of the child's condition.”* Par08_F_Rural |
| Co-operation | **S1** *“I took a photo and in a series of about 3 hours, you could see the breakdown of it [the wound] happening; we were able to use it as a thing, to say this needs urgent attention now…I bumped into one of the doctors and she told the surgeon… I think I showed her the photo of it breaking down and she called the surgeon, and the surgeon stepped in.”* Car01_F56_R |
|  | **S2** *“I think because if you feel under control in control of your own child health you can take a deep breath because if you find working with them [doctors] they're working with me. And I know that they will do all that they can to help to help your child as well.”* Par08_F_Rural |
| Supported Autonomy | **S1 “***So, they’re kind of supporting you in that, or guiding you and that sort of thing. Because I would come back with questions or feedback or, you know, the photographs, and that sort of thing”* Pat02_M48_R |
|  | **S2 “***Apart from that feeling of being proactive, that you are doing something and then this feeling of being sort of backed up by someone that knows about it.”* Pat03_M33_I |
| **Theme: Service & Experience Optimisation (Behavioural)** | |
| Resource Management | **S1** “*You can monitor things yourself. If you notice a significant change in 6 months’ time, because you’re looking at yourself and you can compare it with the original photo that was taken. So, you might go back to the doctor in 6 months, rather than waiting the 12 months for your scheduled check-up.”* Pat02_M48_R |
|  | **S2** “*It [remote diagnosis via images] doesn't make me waste my resources it doesn't waste hospital resources by having to deal with an overly sensitive parent.”* Par10_F_Urban |
| Patient Deviance | **S1** *“I had an experience with one of the patients. I was seeing him for a particular type of rash. I thought it could be something but he was insistent that the rash was something else based on the picture. In tough situations like this, these were the wrong pictures.”* Doc01_F31_SurgicalCare |
|  | **S2** *“[Using photographs] people might have demanded to see a surgeon straight away, or, whatever and I probably just tried to stay calm, and just say, you know, “when you got to me that you just popped in here and have a look at this.”* Par08_F_Rural |
